# Cold Atmospheric Plasma: methods of production and application in dentistry and oncology

**DOI:** 10.1186/2045-9912-3-21

**Published:** 2013-10-01

**Authors:** Clotilde Hoffmann, Carlos Berganza, John Zhang

**Affiliations:** 1 Department of Physiology, Loma Linda University School of Medicine, Risley Hall, Room 223, Loma Linda, CA 92354, USA; 2 Department of Neurosurgery, Loma Linda University School of Medicine, Loma Linda, CA, USA

**Keywords:** Cold Atmospheric Plasma, Dentistry, Oncology, Reactive Oxidative Species, Apoptosis, Senescence, Necrosis

## Abstract

Cold Atmospheric Plasma is an ionized gas that has recently been extensively studied by researchers as a possible therapy in dentistry and oncology. Several different gases can be used to produce Cold Atmospheric Plasma such as Helium, Argon, Nitrogen, Heliox, and air. There are many methods of production by which cold atmospheric plasma is created. Each unique method can be used in different biomedical areas. In dentistry, researchers have mostly investigated the antimicrobial effects produced by plasma as a means to remove dental biofilms and eradicate oral pathogens. It has been shown that reactive oxidative species, charged particles, and UV photons play the main role. Cold Atmospheric Plasma has also found a minor, but important role in tooth whitening and composite restoration. Furthermore, it has been demonstrated that Cold Atmospheric Plasma induces apoptosis, necrosis, cell detachment, and senescence by disrupting the S phase of cell replication in tumor cells. This unique finding opens up its potential therapy in oncology.

## Introduction

**Figure 1:**
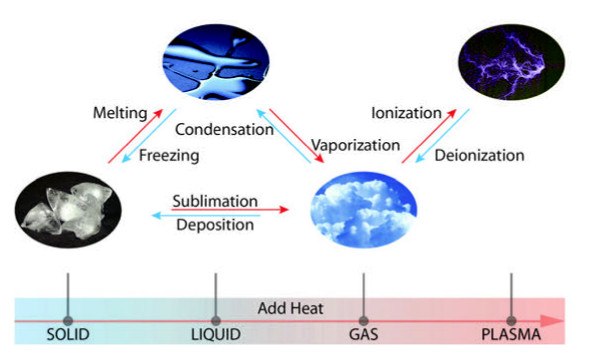
The four states of matter.

William Crookes identified plasma in 1879. 99% of the visible universe is made up of plasma, referred to as the fourth state of matter. The other states of matter are liquid, gas, and solid (Figure 1). Plasma is a partially ionized gas with ions, electrons, and uncharged particles such as atoms, molecules, and radicals. There are two types of plasma: thermal and non-thermal or cold atmospheric plasma. Thermal plasma has electrons and heavy particles (neutrals and ions) at the same temperature. Cold Atmospheric Plasma (CAP) is said to be non-thermal because it has electrons at a hotter temperature than the heavy particles that are at room temperature. CAP is a specific type of plasma that is less than 104°F at the point of application. There are several methods to produce CAP such as Dielectric Barrier Discharge (DBD), Atmospheric Pressure Plasma Jet (APPJ), plasma needle, and plasma pencil. Several different gases can be used to produce CAP such as Helium, Argon, Nitrogen, Heliox (a mix of helium and oxygen), and air. Due to the ability of CAP to deactivate microorganisms, cause cell detachment, and cause death in cancer cells, researchers have been interested in finding uses for CAP in dentistry and oncology.

## Methods of production

Several different types of CAP have been developed for biomedical uses. Energy is needed to produce and maintain plasma. Thermal, electric, or light energy can be used. Usually, the discharge needed to produce CAP is induced electrically. Some methods used to produce CAP include: Dielectric Barrier Discharge (DBD), Atmospheric Pressure Plasma Jet (APPJ), Plasma Needle, and Plasma Pencil.

### Dielectric barrier discharge

In 1857, Siemens was first to conduct experiments on Dielectric Barrier Discharge (DBD). DBD has many applications including: sterilization of living tissue, bacteria inactivation, angiogenesis, surface treatment, and excimer formation [1-12]. The dielectric barrier discharge (DBD) consists of two flat metal electrodes that are covered with dielectric material. A carrier gas moves between the two electrodes and is ionized to create plasma. One electrode is a high voltage electrode and the other is a grounded electrode. High voltages are required to produce the discharge needed to create the plasma. Alternative Current (AC) high voltages generally drive dBD’s with frequencies in the kHz range. The power consumption is between 10 and 100 W [13-16]. There are many variations in the configuration of the electrodes, but the concept behind them all remains the same. For example, some electrodes are cylindrical instead of flat and sometimes the dielectric material covers only one electrode instead of both.

**Figure 2:**
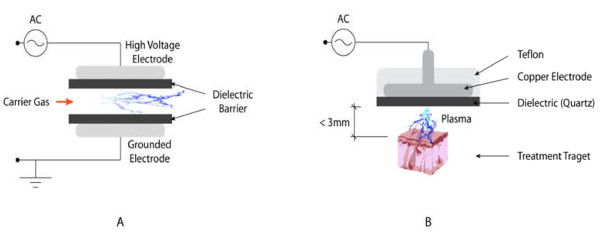
.**Diagram of a Dielectric Barrier Discharge and a Floating Electrode Dielectric Barrier Discharge. A** presents the formation of Plasma by the Dielectric Barrier Discharge (DBD) and **B** presents the Floating Electrode Dielectric Barrier Discharge (FE-DBD).

More recently, Fridman et al. developed the floating-electrode DBD (FE-DBD) [17]. It is similar to the original DBD and consists of two electrodes: an insulated high voltage electrode and an active electrode. The difference between FE-DBD and DBD is that the second electrode is not grounded; it is active meaning that the second electrode can be human skin, a sample, and even an organ. The powered electrode needs to be close to the surface of the second electrode (< 3mm) to create the discharge. It has been used on endothelial cells, melanoma skin cancer, and blood coagulation. It has also been used in living tissue sterilization and in deactivation of *Bacillus stratosphericus* (Figure 2) [18-22]. Plasma jets using a DBD system have also been created [23-25].

### Plasma jet

#### ***Radio frequency plasma jets***

One type of plasma jet, which is employed for bacterial sterilization, is the Atmospheric Pressure Plasma Jet (APPJ) [26]. The APPJ consists of two coaxial electrodes between which a feed gas (mixtures of helium, oxygen, and other gases) flows at a high rate. The outer electrode is grounded while Radio Frequency (RF) power (50-100W) at 13.56 MHz is applied to the central electrode that creates a discharge. The reactive species produced exits the nozzle at high velocity and arrives to the area that is to be treated. APPJ has been used for the inactivation of several micro-organisms [27-35].

**Figure 3:**
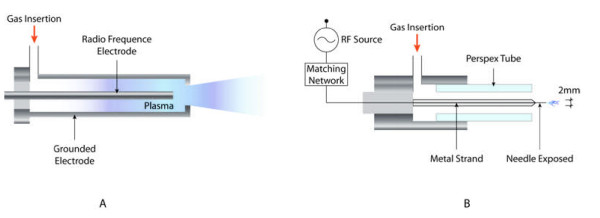
.**Diagram of a Atmospheric Pressure Plasma Jet and a Plasma Needle. A** presents a schematic of the APPJ created by Schütze et al in 1992 and **B** presents a schematic of the plasma needle created by Stoffels et al in 2004.

Koinuma et al. developed the earliest RF cold plasma jet in 1992 [36]. The cathode is a needle electrode made of tungsten or stainless steel with a 1 mm diameter connected to a RF source (13.56 MHz). The needle electrode lies within a quartz tube whereas the anode electrode is grounded. Depending on the application, helium or argon were mixed with various gases. This group published several papers describing its variants and applications of the plasma jet [37-43]. In 2002, Stoffels et al. created a miniature atmospheric plasma jet that they called plasma needle [44] and created a new version in 2004 [45]. In the former version, the needle was enclosed in a box and as a result, the samples had to be placed inside of the box to be treated. In the new version, the plasma needle consists of a 0.3 mm metal strand diameter with a sharpened tip inside of a Perspex tube. The length of the entire needle is 8 cm and 1.5 cm remains uncovered by the Perspex tube. The gas used most frequently is Helium due to its high thermal conductivity. The gas is then mixed with air at the needle tip where a micro discharge is created. Gases other than Helium are also used [46]. The diameter of the plasma glow generated is 2 mm. Microplasma is created when RF power at 13.05 MHz ranging between 10 mW and several watts is applied to the needle. Its small size enables it to be used to treat small areas where accuracy is required like in dentistry [47-52]. It has also been used to deactivate *E. Coli* (Figure 3) [53].

#### ***Pulsed direct current-driven plasma jets***

**Figure 4:**
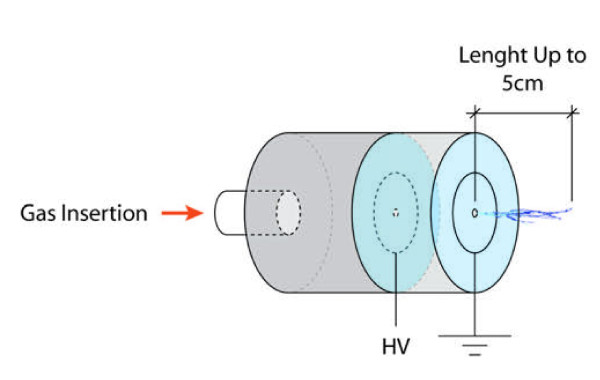
A schematic of the plasma pencil created by Laroussi et al.

Laroussi et al. developed a miniature jet that they called plasma pencil [54]. It consists of a dielectric cylindrical tube of 2.5 cm in diameter where two disk electrodes of the same diameter as the tube are inserted. The two electrodes are separated by a gap (the distance can vary from 0.3 to 1 cm) and consist of a thin copper ring attached to a dielectric disk. To create the plasma, sub-microsecond high voltage pulses are applied between the two electrodes while a gas is injected through the holes of the electrodes. When the discharge is created, a plasma plume is launched through the hole of the outer electrode into the air. Because the plasma plume (up to 5cm in length) remains at low temperature (290K), it can be touched safely. The electrical power is supplied to the electrodes by a high voltage pulse generator. The high voltage is supplied to the pulse generator by a DC voltage supply with variable output. The plasma pencil has been used in the treatment of *E. coli*, Leukemia cells, and *P. Gingivalis*[55-57]. Forster et al., Zhang et al., and Wash et al. developed a plasma jet using a DBD configuration (Figure 4) [58-60].

### Different components of plasma involved in sterilization

Laroussi et al. first demonstrated in 1996 that glow discharge plasma generated at atmospheric pressure is a very effective sterilization agent [61]. The reactive species, charged particles, and UV photons are said to be the major components involved in sterilization of a wide range of gram-positive bacteria, gram-negative bacteria, spores, biofilms, viruses, and fungi [62-64].

#### ***Effect of reactive oxidative species***

According to several authors, reactive neutral species such as Oxygen, Hydroxyl Radicals, and Nitrogen Dioxide play the main role in the use of plasma for sterilization purposes. In 1999, Herrmann et al. used APPJ with and without oxygen. They observed that the D value (the time needed to kill 90% of the microorganisms) was higher when oxygen was absent [27]. Moreau et al. concluded that oxygen played the main role in the sterilization of *Bacillus Subtilis*[65] whereas Richardson et al. also observed that adding oxygen to the discharge gas helium made the device more effective at killing bacteria [66]. Kuzmichev et al. concluded that the best bactericidal effects were found using moistened oxygen and air.

Laroussi and Leipold used different gases to deactivate Bacillus spores: either pure helium, a mix of 97% helium/ 3% of oxygen, or air. They observed that the use of pure helium resulted in a D value of over 20 minutes, the use of the mixture of helium and oxygen resulted in a D value of 10 minutes, and the use of air resulted in a D value of 20 seconds [67]. The oxygen species were found to play the major role in the sterilization process due to their strong oxidative effects on the outer structures of cells [68].

Reactive Oxidative Species (ROS) was found to be the major mechanism involved in the deactivation of bacteria by the FE-DBD plasma [69]. They observed that plasma generates ROS that causes morphological changes of *E. coli*, depolarization of the membrane, lipid peroxidation, and DNA damage in a dose dependent manner. In this study they also used ROS scavengers and found no inactivation of *E. coli* after the plasma treatment. This confirms that ROS is the major component involved in the sterilization process.

#### ***Effect of charged particles***

Kelly-Wintenberg et al. employed an atmospheric pressure glow discharge for the inactivation of Gram-negative *E. coli* and used transmission electron microscopy (TEM) to visualize the plasma-induced physical damage to the microorganism. Plasma exposure rapidly disrupts the cell wall and leads to a release of cellular contents in the surrounding medium [70].

Mendis et al. [71] and Laroussi et al. [72] suggested that charged particles can play a significant role in the rupture of the outer membrane of bacterial cells. They showed that the electrostatic force caused by charge accumulation on the outer surface of the cell membrane could overcome the tensile strength of the membrane and cause its rupture. Nevertheless, it is more likely to occur for gram-negative bacteria because of its irregular cell surface. Laroussi et al. confirmed this by not observing any rupture of the cell of the gram-positive *B. Subtilis*. Furthermore, Fridman et al. showed that charged particles play an essential role in sterilization, especially when the plasma is in direct contact with the microorganisms. They observed that a direct application of plasma resulted in better sterilization efficacy. They concluded that it might be possible that charge-induced mechanisms contribute to the sterilization process in direct plasma exposure [73]. Stoffels et al. confirmed that charged particles play an important role [74].

#### ***Effect of UV radiation***

According to the literature, the role of UV radiation in the sterilization process is still unclear. The presence of UV radiation in the plasma strongly depends on the operating pressure. Vacuum plasma at very low pressure discharges can produce UV radiation in the range of wavelengths known to be involved in sterilization (200–290 nm) [75].

Nevertheless, at atmospheric pressure, plasma does not produce UV radiation in adequate wavelengths to produce sterilization. In 1996, Laroussi et al. compared the efficacy between sterilization with UV radiation produced by a low-pressure mercury vapor lamp versus glow discharge plasma at atmospheric pressure. They concluded that UV radiation was not the first agent involved in the sterilization process at atmospheric pressure [61]. Choi et al. and Laroussi et al. measured the wavelengths of UV radiation produced at atmospheric pressure. Choi et al. treated samples with a DBD operated in air at atmospheric pressure and did not observe any UV radiation below 290 nm [76]. Laroussi et al. did not observe any UV radiation in the wavelength of 200–285 nm after using the flowing afterglow of a DBD in air at atmospheric pressure on spores of *B. genus*[68].

Kelly-Wintenberg et al. exposed several microorganisms to an atmospheric pressure glow DBD in air. According to the authors, the time needed to deactivate the microorganism was the same if the samples were in opaque bags or not, negating that deactivation was due to UV radiation [77]. Herrmann et al. treated *Bacillus globigii* with an atmospheric pressure plasma jet operating in helium/oxygen mixtures and blocked the reactive species produced with a quartz window in order to allow only UV radiation to be in contact with the spores. They did not observe any significant decrease in the number of bacteria after treatment [27]. Birmingham et al. tested a plasma blanket and noticed that the plasma blanket does not generate sufficient photons of the appropriate wavelength and therefore concluded that the deactivation of the bacterial spore was not the result of the UV radiation [78]. In the plasma needle created in 2004 by Stoffels et al., UV emission was quantified between 250 and 400 nm with the highest intensities between 305 and 390 nm. At these wavelengths, the damage to cells and tissues is limited [45]. Kostov et al. also concluded that UV radiation does not play any significant role in the sterilization process [79]. The preponderance of the studies suggests that UV radiation does not contribute significantly to the sterilization process.

Nevertheless, some authors do mention the possible role of UV radiation in plasma sterilization at atmospheric pressure. Trompeter et al. [80] and Heise et al. [81] both used argon plasma and concluded the inactivation of spores was due to UV radiation. Park et al., Lee et al., and Boudam et al. also claimed that UV radiation has a main role [82-84]. Further studies are required to investigate and clear up these controversies in the literature.

### CAP in dentistry

**Figure 5:**
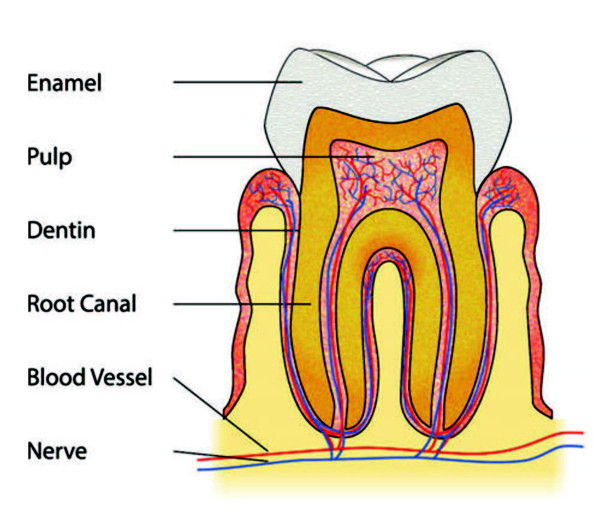
**The different components of a tooth**[153]**.**

The mouth is a microbial habitat with over 700 species that live in harmony with the human body [85]. However, periodontal disease and caries are the two most common diseases in dentistry. Every year, $60 Billion is spent in the United States to treat dental disease. Dental caries are defined as the localized destruction of tooth tissue by the acids produced by bacteria. [86]. Caries start with small demineralization areas under the enamel. The demineralization can progress through the dentine and to the pulp (Figure 5). *S. mutans* is one of the major causes of caries [87]. Before filling cavities, necrotic, infected, and demineralized tissue is removed by using ozone treatment, mechanical drilling, or laser techniques [88-95]. Unfortunately, these methods can be destructive as they might remove an excess of healthy tissue to make sure that the cavity is bacteria free. Periodontal disease is related to dental plaque, which is a complex oral biofilm with several microbial species organized in communities [96]. It leads to the detachment of the gum from the tooth as a result of inflammation, which is the body’s natural response to dental plaque. Several authors have been studying possible use of CAP on dental bleaching, dental disinfection, biofilm removal, instrument sterilization, and composite restoration (See list of different uses of CAP in dentistry section).

#### ***List of different uses of CAP in dentistry***

Deactivation of Biofilms-

o S. mutans [97]

o B. cereus and G. Stearothermophilus [98]

o L. acidophilus and S. mutans [99]

o P. Gingivalis [57]

o S. mutans [100]

o Root canal disinfection [101]

o E. coli, L. casei, S. mutans and C. albicans on agar and dentine plates [102]

o E. faecalis in the root canal [103]

o Ex-vivo biofilms on root canals of extracted teeth [50]

Tooth Bleaching-

o Hydrogen Peroxide + CAP enhanced the tooth bleaching [104-109]

o CAP + saline [110]

o Carbamide Peroxide + CAP [111]

o Plasma plume + 36% H_2_O_2_ gel on extracted teeth [112]

Instrument Sterilization-

o Removal of biofilms on microstructures titanium [113,114]

o Dental instruments [115]

o Ti discs inoculated with biofilms [116]

Composite Restoration-

o CAP treatment increases dentin/adhesive interfacial bonding [117]

o CAP treatment improves the tensile-shear bond strength between post and composite [118]

#### ***Use of CAP on oral pathogens***

A promising application of CAP in dentistry is the disinfection of dental cavities due to its high efficiency at deactivating biofilms. It could offer a less destructive method to prepare dental caries for filling. Since it operates at room temperature, it does not cause any pain or destruction of the tissue. Cold Atmospheric Plasma could also be used for the treatment of periodontal disease based on its microorganism deactivation property.

CAP has been shown to be effective at deactivating biofilms. Goree et al. investigated using a plasma needle to kill *S. mutans*, which is the main microorganism causing dental caries [97]. They observed that the plasma needle could kill these bacteria after 10 seconds of treatment. They concluded the plasma needle could provide an attractive alternative for dental clinical treatment. Morris et al. used CAP to deactivate *Geobacillus stearothermophilus* and *Bacillus cereus* microorganisms [98]. *B. cereus*, a gram-positive microorganism, has been associated with periodontal disease and food poisoning. *G. stearothermophilus* is used as a biological indicator of treatment efficacy in sterilization studies. Both of the microorganisms were in vegetative cells and spores and were treated either with direct or indirect plasma. They concluded that cold plasma is effective in killing *B. cereus* vegetative cells and spores at various time points. *G. stearothermophilus* vegetative cells were killed with direct and indirect exposure to plasma. In 2011, Yang et al. used a cold atmospheric argon plasma brush to deactivate oral bacteria of *Streptococcus mutans* and *Lactobacillus acidophilus*[99]. They observed that the argon plasma brush was effective at killing bacteria. Between 11 and 15 seconds (depending on the bacterial supporting media) was needed to deactivate *S. mutans*. A little more time was needed to deactivate *L. acidophilus*: up to 5 minutes, depending on the bacteria supporting media.

Mahasneh et al. used Low Temperature Atmospheric plasma to kill *Porphyromonas gingivalis*, which is a periodontal pathogen associated with periodontal disease [57]. The plasma pencil created by Laroussi et al. was used for the experiment. They observed a significant increase in a dose dependent manner in the inactivation of *P. gingivalis* in the treatment group compared with control. Kang et al. used RF atmospheric plasma to inactivate *Streptococcus mutans*[100]. The gas used was a mix of Argon and Hydrogen Peroxide. They observed that the inactivation efficacy was highly dependent on the Hydroxyl radical concentration. Moreover, by adding hydrogen peroxide to the gas, they decreased the ozone formation that is naturally formed in CAP. Ozone has bactericidal properties, but is toxic which is a disadvantage for the use in the clinic.

CAP was also effective at destroying biofilms either on root canals or on dental slices. Jiang et al. developed a plasma plume at room temperature [102]. They used it to disinfect root canals from extracted human teeth. Two teeth were placed at a distance of 5 mm from the plasma nozzle. One of them was exposed to the helium/oxygen plasma for 5 minutes, whereas the other one was exposed to the same helium/oxygen flow for five minutes, but without plasma. They observed better results in the reduction of the biofilms in the tooth treated with plasma compared with control. Nevertheless, the plasma failed to reach the lower zone of the tooth. The authors explained it by the fact that the plasma plume did not have the optimal width and length to effectively treat the lower zone.

Rupf et al. used a CAP jet to kill adherent oral microbes, known to cause dental carries [102]. The gas mixture used was Helium, Oxygen, and Nitrogen. Four microorganisms were used: *E. coli, L. casei, S. mutans, and C. albicans*. These microorganisms were placed on agar plates and on Dentin slices. The plasma treatment showed antimicrobial efficacy against all of the organisms. The antimicrobial efficacy was better on agar plates than on dental slices. Moreover, *S. mutans*, a gram-positive bacteria, showed the strongest resistance to plasma-jet treatment.

Lu et al. used a plasma-jet device, which could generate plasma inside the root canal [103]. They used it on *Enterococcus faecalis*, which is one of the most important bacteria causing failure of the root-canal treatment. Owing to its low temperature, the plasma could be touched and placed into the root canal without any pain. A mix of Helium & Oxygen was used. They observed that the plasma jet was efficient at deactivating *Enterococcus faecalis*.

In 2013, Schaudinn et al. used a plasma needle to eliminate *ex vivo* biofilms on root canals of extracted teeth [50]. They divided the teeth into three groups: treatment with the plasma needle, treatment with 6% sodium hypochlorite (an antiseptic), and control. They concluded that the plasma needle was effective at killing biofilms in extracted teeth. However, using 6% sodium hypochlorite is more efficient.

#### ***Tooth whitening***

Researchers have been interested in the use of CAP in tooth whitening. Hydrogen peroxide is currently used to whiten teeth [104]. Hydroxyl radicals generated from Hydrogen peroxide play the main role in tooth bleaching [105]. Some researchers looked for an alternative treatment and found CAP to be an interesting candidate. They either used it in complement with the hydrogen peroxide treatment or alone. Lee et al. used an atmospheric pressure plasma jet for tooth bleaching [106]. The carrier gas used was Helium. 28 extracted teeth were used for the experiment. All of them were cut in half longitudinally and all the pieces were placed in two groups. The dentin and the tooth surface of the treatment group received H_2_O_2_ + Plasma for 10 minutes while the dentin and the tooth surface of the control only received H_2_O_2_ for the same amount of time. The results showed a three fold improvement in tooth bleaching of the treatment group compared with control. The greater efficacy of tooth bleaching in the treated group compared with control was reported to be due to both the removal of the tooth surface protein and the double concentration of Hydroxyl radicals.

Lee et al. also investigated bleaching of teeth stained with coffee or red wine. Using plasma with hydrogen peroxide improved the bleaching efficacy by a factor of 3.7 for teeth stained with red wine and 3.1 for teeth stained with coffee compared with using hydrogen peroxide alone [107]. Sun et al. also concluded that using plasma with hydrogen peroxide enhanced tooth whitening compared to hydrogen peroxide alone [108]. Pan et al. created a new method of tooth whitening by using a Cold Plasma Microjet driven by Direct Current at Atmospheric Pressure [109]. 60 teeth were chosen and were randomly divided into three different groups. In the first one, the teeth were exposed to a saline solution and airflow for twenty minutes. In the second group, the teeth were exposed to the plasma and saline solution for twenty minutes. In the last group, the teeth were exposed to hydrogen peroxide gel at room temperature for the same duration of time. They observed that the whiteness of the teeth of the plasma treated group was significantly improved compared to the first and the last group. They suggested that the Reactive Species formed at the plasma-liquid-tooth interface was the cause for greater tooth whitening.

Park et al. demonstrated the effect of CAP on an intracoronal tooth stained with blood [110]. They extracted single root human teeth and cavities were created artificially. The teeth were then artificially stained by hemoglobin-rich hemolysed blood. Two groups were used. The control group was treated with 30% hydrogen peroxide in the pulp chamber for 30 min and the experimental group was treated with 30% hydrogen peroxide with CAP. The bleaching efficacy of the treated group was approximately 2 fold better than that of the control group.

Tooth bleaching agents are mostly based on hydrogen peroxide and carbamide peroxide where light sources can be used in combination. Light sources can increase the effectiveness of the tooth whitening. Nam et al. used a Plasma jet on forty extracted human molar teeth with intact crowns [111]. The forty teeth were randomly divided into four groups (n=10) and were treated with Carbamide peroxide + CAP, Carbamide peroxide + Plasma Arc Lamp (PAC), Carbamide peroxide + diode laser, or Carbamide Peroxide alone (control). They observed CAP was the most effective at bleaching teeth. Moreover, they observed that CAP does not damage the tooth due to its low temperature.

Laroussi et al. used a plasma plume on thirty extracted human teeth randomized into two groups: Group I received plasma plume + 36% H_2_O_2_ gel for 10, 15 and 20 min and Group II received 36% H_2_O_2_ gel only for the same time duration [112]. They observed a statistically significant increase in the whitening of the teeth after exposure to CAP + 36% H_2_O_2_ gel, compared with 36% H_2_O_2_ only, in the 10 and 20 min groups. The temperature in both treatment groups remained under 80°F throughout the study, which is below the thermal threat for vital tooth bleaching.

#### ***Sterilization of dental instruments***

Autoclaves and UV sterilizers are currently used to sterilize dental instruments. To develop a dental sterilizer that can sterilize most materials including metals, rubber, and plastics, researchers have investigated CAP as a universal sterilizer. Rupf et al. employed a CAP jet to remove biofilms on micro-structured titanium [113]. They created biofilms by intraoral exposure on micro-structured titanium discs. A mini plasma device 1.5 cm wide and 2.5 cm tall was used with helium as the carried gas. By using fluorescence microscopy and Scanning Electron Microscopy (SEM) they observed a complete disinfection of the biofilms with a single plasma treatment. Nevertheless, a combination of plasma with air/surface spray resulted in a complete biofilm removal. Using an extra plasma treatment at the end even increased the probability of the complete removal of the biofilm. They also showed that plasma was superior to chlorhexidine in biofilm removal.

Koban et al. used CAP on biofilms of *Streptococcus mutans* and saliva multispecies grown on titanium discs in vitro. They compared the efficacy of sterilizing the disks with CAP and sterilizing with chlorhexidine digluconate [114]. In contrast to Chlorhexidine digluconate (CHX), a mouth rinse solution used in dental clinics, treatment with CAP was very effective against S. mutans and multispecies saliva biofilms.

Sung et al. also used CAP to assess the sterilization effect on dental instruments [115]. They inoculated *B. subtilis* and *E. coli* on diamond burs and polyvinyl siloxane materials. Then they exposed them to the plasma for a different length of time (from 30 to 240 seconds). They compared the plasma device efficacy with the UV sterilizer. The plasma device decreased the colony-forming unit (CFU) for both *E. coli* and *B. subtilis* significantly on both diamond burs and polyvinyl siloxane materials. The atmospheric pressure of non-thermal air plasma showed better sterilization rates than the UV sterilizer.

Idlibi et al. treated Ti discs inoculated with biofilms with CAP [116]. The biofilms were divided randomly among the following treatments: CAP, diode laser, air-abrasion, and chlorhexidine. They observed CAP treatment decreased the quantity of the biofilms the most among all the treatments. Nevertheless, CAP did not succeed in completely removing the biofilms.

#### ***Dental composites***

Dental composites are currently used to fill cavities. Some researchers investigated CAP in composite restorations. The plasma generates reactive species that arrive on the surface of the composite resulting in both microstructural and surface chemistry modifications that improve adhesive bonding. They observed plasma treatment increases bonding strength at the dentin/composite interface that enables it to last longer on teeth.

Ritts et al. investigated a non-thermal atmospheric plasma brush on dental composite restoration [117]. They observed that atmospheric cold plasma brush (ACPB) treatment could modify the dentin surface and increase dentin/adhesive interfacial bonding. Yavrich et al. studied the effects of plasma treatment on the shear bond strength between fiber reinforced composite posts and resin composite for core buildup and concluded that plasma treatment appeared to increase the tensile-shear bond strength between post and composites [118].

### Effects of CAP on malignant cells

#### ***CAP and mammalian cells***

Few studies have been performed on the effect of CAP on eukaryotic cells thus far. Eukaryotic cells are defined as cells where the genetic material is inside the nucleus. Some researchers observed either cell detachment, decrease of cell migration, apoptosis, or necrosis on several types of cells depending on the power and the time of exposure to plasma. Necrosis is defined as an unprogrammed death of cells in living tissue. This leads to inflammation by releasing intracellular content. In contrast with necrosis, apoptosis is a programmed cell death process resulting in no inflammation. Different groups have conducted *in vitro* experiments with fibroblasts, endothelial cells, ovarian cells, human hepatocytes, and smooth muscle cells. Stoffels et al. used a plasma needle on Chinese Hamster ovarian cells and observed different results depending on the power and the time of exposure. For exposure times longer than 10s and powers higher than 0.2W, necrosis was observed. With lower doses of exposure to the plasma, apoptosis was observed. With power level at about 50 mW and an exposure time of 1s, the cells detached from the sample without undergoing apoptosis [119]. Yonson et al. [120] also showed detachment of human hepatocytes (HepG2) after CAP treatment. Shashurin et al. used a plasma jet on fibroblast cells and observed cell detachment at medium plasma treatment levels [121]. Kieft et al. [122] induced apoptosis in 3T3 mouse fibroblast cells and in another study they used a plasma needle for treatment of mammalian endothelial and smooth muscle cells. At lower doses cell detachment was observed while at higher doses necrosis was observed [123]. Some researchers observed that CAP decreases cells migration of both fibroblasts and epithelial cells by increasing integrin activation [124].

#### ***CAP and malignant cells***

Because of the effect of CAP on mammalian cells, researchers have been interested in using it on malignant cells also. The conventional therapies for cancerous diseases are based on removal of the tumor, chemotherapy, or radiation. Nevertheless some cancers remain hard to eradicate. *In-vitro* and *in-vivo* studies have been performed on the efficacy of CAP at killing cancer cells. The results of the pilot studies performed by several research groups confirmed that treatment with low-temperature plasma is able to induce several modes of cell death including apoptosis and necrosis. They also noticed decreased cell migration and induction of senescence in cancer cells. Regarding the mechanism of the Atmospheric Pressure Plasma therapy on cancer cells, the hypothesis is that the ROS plays the main role. ROS are well known to be harmful to cells inducing apoptosis, senescence, or cell cycle arrest [125]. Sensenig et al. proposed that ROS is the mechanism through which CAP induces apoptosis [126].

#### ***Effect of CAP on various cancers***

A few *in vitro* and *in-vivo* studies have been published by researchers regarding CAP use in cancer. In an *in vitro* study, Fridman et al. used a FE-DBD plasma treatment to treat Melanoma cancer cells [20]. They observed either apoptosis or necrosis depending on the dose of the treatment. Melanoma cells, treated by plasma at low dose, developed apoptosis several hours post treatment. At higher dosage, melanoma cells developed *necrosis.* Apoptosis was also observed on cultured human breast cancer cells treated with a pulsed atmospheric pressure plasma jet used with Heliox [127]. At low plasma dosage apoptosis was observed while at a higher dose, necrosis was observed. Thiyagarajan et al. observed that CAP can cause cell death in leukemia cancer cells (THP-1 cells), and there is a dose dependent response in the induction of cell death. They also observed that higher treatment doses cause necrosis, while lower treatment doses induce apoptosis [128]. In 2012, Partecke et al. observed Tissue Tolerable Plasma (TTP) treatment significantly induces apoptosis in pancreatic cancer cells *in vitro*, with treatment duration of 10 seconds showing the strongest effect [129].

Walk et al. used a CAP on neuroblastoma cells in vitro [130] and concluded that CAP decreases metabolic activity, induces apoptosis, and reduces numbers of viable cancer cells in direct proportion to the duration of treatment. Kaushik et al. used an Atmospheric Pressure non-thermal plasma jet on T98G brain cancer cells [131]. They observed the mortality percent of T98G cells directly depends on exposure time. As plasma exposure increases, the plasma treatment increases cell death and inhibits the colony formation capability of the T98G cell population. They observed that plasma treatment inhibits the colony formation capabilities of T98G cells at all doses.

Glioblastoma is the most aggressive brain tumor in adults. Therapy with Temozolomide (TMZ) is efficient only when patients have methylation of the MGMT gene in the tumor. Köritzer et al. used plasma produced on a Surface Micro-Discharge (SMD) electrode on the human glioblastoma cell lines LN18, LN229 and U87MG [132]. The cell lines U87MG and LN229 do not express the MGMT protein, while the cell line LN18 expresses the MGMT protein. TMZ was also used as a treatment on the human glioblastoma cell lines either alone or in combination with CAP. They observed TMZ was only efficient on the cell lines with MGMT when used alone. They observed that previous CAP treatment restores the sensitivity of TMZ resistant glioma cells. 60 seconds of CAP treatment combined with 100 mM or 200 mM TMZ showed a statistically significance increase in inducing a cell cycle arrest in G2/M phase compared with TMZ treatment alone.

Laroussi et al. used a plasma pencil on non-adherent leukemia cancer cells and Helium was the carrier gas used [133]. CCRF-CEM cells, which are non-adherent leukemia cancer cells, were suspended in media solution and treated with CAP for 0–10 minutes. They observed a dose dependent response in the induction of cell death. They suppose that a longer exposure to plasma results in an increase in the reactive species formation. The delayed effect of plasma exposure on leukemia cells might be attributed to the initiation of an intracellular signaling cascade that leads to programmed cell death.

Some researchers have studied the effect of CAP on the invasion activity in colorectal cancer [134]. They concluded that CAP significantly inhibited cell migration and invasion in SW480 colorectal cancer cells. However, the best results were when they were treated with a mix of Helium plus Oxygen compared to control or Helium only. They also observed that increasing plasma voltage improves the results.

Cold atmospheric plasma can be used as a new strategy to induce senescence in melanoma cells [135]. Some researchers used a ‘miniFlat-Plaster’ that uses the flexible and scalable Surface Micro Discharge (SMD) technology for plasma production in air. Melanoma cells were treated for either one or two minutes. Two minutes of CAP treatment resulted in about 50% apoptosis in melanoma cancer cell lines. On the contrary, 1 min of CAP treatment was not enough to induce apoptosis, but it created senescence (a permanent cell cycle arrest, considered as a good mechanism which prevents aged or abnormal cells from expansion) and as a result stopped proliferation. Kim et al. used Atmospheric non-thermal plasma to treat HCT-116 colorectal cancer cells and also observed CAP induced cell growth arrest and apoptosis. Moreover, plasma reduced cell migration and invasion activities [136].

#### ***In-vivo studies on the use of CAP in cancer***

Walk et al. performed an in vivo study of neuroblastoma cells injected in mice [130]. Mice were injected with Neuro2a cells and treated with CAP. 7 treated mice received 5 min of CAP while 7 control mice received no therapy after inoculation. CAP initially ablated the tumors. Although tumors recurred in some mice, their growth rate was decreased and median survival of the mice in the treatment group was almost two fold from 15 to 28 days. CAP treatment resulted in a dramatically improved survival compared to the control group.

In another in-vivo study, Kim et al. demonstrated no initial reduction in melanoma size, but did show CAP's ability to inhibit tumor growth in mice [137]. Female mice between 6 and 8 weeks old were subcutaneously injected with B16F0 cells. After tumors reached a size of about 40 mm^3^, the mice were treated with a microplasma for 5 seconds each either one time or four times for four consecutive days. They then measured the length and width of the tumors every 2 to 3 days and they calculated the tumor volume. They observed no antitumor effect for the one-time treatment. However, the four-time treatment was effective at inhibiting tumor growth (Table 1).

**Table 1:** In vitro and in vivo studies performed in Oncology with CAP

**Studies performed in Oncology**	**Types of cancer**
In vitro studies	Melanoma cells [20], Human Breast cancer cells [127], Leukemia cells (THP-1 cells) [128], Pancreatic cancer cells [129], Neuroblastoma cells [130], T98G brain cancer cells [131], human glioblastoma cell lines LN18, LN229 and U87MG [132], CCRF-CEM cells (non-adherent leukemia cells) [133], Colorectal cancer cells (SW480 cells) [134], Melanoma cells [135], Colorectal cancer cells (HCT-116 cells) [135], lung cancer (SW900) cell lines and murine melanoma cells [143], TC-1 lung carcinoma cells [145], Murine melanoma B16F0 tumor cells [144], B16 cancer cells and COLO320 cancer cells [144], Lung cancer cell lines (H460 and HCC1588) [145]
In vivo studies	Pancreatic tumor [129], Neuroblastoma [130], Melanoma [137], Glioma [138], Bladder cancer tumor and melanoma [139]

Vandamme et al. used FE-DBD plasma on U87-bearing mice [138]. They started the treatment when the tumor reached 150 ± 50 mm^3^ corresponding to Day 0. Mice received a daily plasma treatment for 6 minutes for five consecutive days. At day 6 they measured the tumor volume and observed a significant reduction of 56% for the treatment group compared with the control. They also performed a bioluminescence imaging (BLI) of the tumor on Day 0 (D0) and Day 6 (D6). They calculated the ratio D6/D0 of BLI intensity, corresponding to the tumor activity between the beginning and the end of the treatment. They observed in the control group a 24-fold BLI intensity increase, whereas in the treatment group, BLI intensity only increased sevenfold. They also evaluated the long-term effect of the plasma treatment. After plasma completion, tumors started to grow again, but slowly compared with the control group. They also observed a decrease in mortality for the treatment group of 58%. These results showed significant anti-tumor property of the CAP treatment.

Keidar et al. applied a plasma jet to 10 nude mice bearing subcutaneous bladder cancer (SCaBER) and to 8 mice with B16 melanoma cells. In the bladder cancer group, they observed that a single treatment with CAP of 5 minutes led to tumor ablation. They also observed that tumors of about 5mm in diameter are ablated after 2 min of single time plasma treatment, whereas larger tumors decreased in size. Moreover, the ablated tumors did not grow back, while partially removed tumors started growing back a week after treatment without reaching the original size even after 3 weeks post-treatment. They also observed good outcomes for the mice inoculated with melanoma. After the treatment with CAP for five minutes, they observed the tumor growth rates were markedly decreased and the median survival was 33.5 days while it was of 24.5 days for the control group [139].

Partecke’s *in-vivo* experiment using TTP induced apoptosis only in the top cell layers of pancreatic tumor showing a depth of effective tissue penetration of up to 60μm [129]. Some improvement needs to be done to enable plasma to reach deeper in the tumor.

#### ***CAP in cancer therapy conjugated to gold nanoparticles***

Kim et al. created a novel approach to treat cancerous cells by incubating cancer cells with gold nanoparticles making them more vulnerable to CAP treatment. They bound Gold nanoparticles (GNP) to G361 melanoma skin cancer cells with an anti-FAK antibody. FAK antibody is a protein overexpressed in G361 melanoma cells compared to normal tissues. They observed a five-fold improvement of melanoma cell death over using plasma alone by using air plasma with gold nanoparticles bound to anti-FAK antibodies [140]. Later, they investigated the effect of GNP conjugates with anti-EGFR antibody and anti-TFR antibody treated with CAP for the selective treatment of cancerous cells [141]. Epidermal growth receptor (EGFR) and transferring receptor (TFR) are over-expressed in several oral cancer cells. Therefore, anti-EGFR antibody and anti-TFR antibody were conjugated to GNPs for targeting oral cancers. They observed a significant improvement of oral carcinoma cell death over using plasma alone by using CAP with bound nanoparticles to anti-EGFR antibody or anti-TFR antibody.

#### ***CAP selectivity of cancer cells***

Despite the good results observed on in vitro and a few in vivo studies, more work is required to make CAP useful in the clinic. Finding a therapy for cancer remains challenging because it has to selectively attack only the cancer cells and let the normal cells live. Some researchers found that the cancer cells are more sensitive to CAP treatment than normal cells, which could make CAP an ideal cancer therapy.

Volotskova et al. showed that the cancer cells are more susceptible to the effects of CAP because a higher percentage of cells are in the S phase of the cell cycle [142]. The cell cycle is defined as a series of events that takes place in a cell leading to its division and replication. The cell cycle consists of four different phases: G1, S (Synthesis of DNA), G2 (Interphase) and M phase (Mitosis). Between S and G2 phases and between G2 and M phases we can find checkpoints that check if the processes at each phase of the cell cycle have been accurately completed before progressing into the next phase. Volotskova et al. found that CAP delayed progression of skin cancer cells by hindering them at the checkpoint between G2 and M phases. It correlated with the increase of cH2A.X that is a marker showing damage in the S phase of the cycle.

Keidar et al. used CAP *in vitro* on normal human Bronchial epithelial cells (NHBE), lung cancer (SW900) cell lines, murine melanoma cells, and primary macrophages. They observed cell detachment of 60–70% of SW900 cancer cells treated with plasma, while no detachment was observed in the treated zone for the normal human Bronchial epithelial cells (NHBE). Plasma treatment leads to a significant reduction in SW900 cell number, whereas NHBE cell count remains almost the same. Concerning the murine macrophages and B16 melanoma cells, they observed plasma selectivity for murine melanoma cancer cells while murine macrophages were not affected [139].

Kim et al. used a microplasma jet device on mouse TC-1 lung carcinoma and CL.7 fibroblast cells. They observed more apoptotic activity in TC-1 lung carcinoma cells compared with the CL.7 fibroblast cells treated with the same dosage and same duration. They concluded that the TC-1 tumor cells are more sensitive to plasma treatment than CL.7 fibroblast cells under these experimental conditions. They even noticed for certain plasma dose conditions, the microplasma jet induced only apoptosis of the TC-1 lung carcinoma cells. This microplasma could be used to selectively kill TC-1 lung carcinoma cells [143]. In another study, they also used a micro-plasma to treat both Murine melanoma B16F0 tumor cells and murine fibroblast CL.7 cells for 0–20 seconds respectively [144]. They observed the murine melanoma tumor cells were more sensitive to plasma treatment than murine fibroblast cells under specific plasma dose conditions. The plasma treatment induced more apoptosis in B16F0 tumor cells than in CL.7 cells when the treatment was lower than 20 seconds.

Gweon et al. used a microplasma jet on both metastatic cancerous SK-HEP-1 and normal THLE-2 cells for a duration of two minutes with Helium as a carrier gas [145]. They observed that the cancer cells had a better ability to detach compared to normal cells after treatment. According to the biochemical and biophysical assays, cancer cells seemed to have weaker adhesion and different responses against plasma treatment compared to the normal cells.

Georgescu et al. observed no apoptosis in macrophage cells treated with CAP while apoptosis was observed on B16 cancer cells and COLO320 cancer cells [146]. Amdt et al. also observed that normal melanocytes are less sensitive to CAP therapy in comparison with tumor cells derived from primary or metastatic melanomas [135].

Panngom et al. treated Lung cancer cell lines (H460 and HCC1588) and lung normal cell lines (MRC5 and L132) with non-thermal DBD plasma [147]. They observed higher apoptotic cell death in lung cancer cell lines than that in lung normal cell lines treated with plasma.

#### ***Molecular mechanism of the action of CAP on cancer cells***

Tuhvatulin et al. have been interested in the mechanism involved in the cell death after plasma exposition. They observed that CAP treatment of human colon carcinoma cells (HCT116) induces activation of protein p53, known to initiate cell death via the p53-dependant pathway. They found that the activation of caspase 3 depends on the presence of p53. They concluded that treatment of human colon carcinoma cells by CAP results in apoptosis dependent of p53 [148]. Nevertheless, more studies need to be performed regarding the type of damage in cells resulting in p53 activation.

Yan et al. observed plasma treatment increases the percentage of apoptotic cells being associated with cell cycle arrest at the G2/M phase. They found the expression of the p21 CDK inhibitor (cell cycle inhibitor) and the protein p53 are increased [149].

Vandamme et al. treated human glioblastoma (U87MG) and human colon carcinoma (HCT-116) cells with CAP. They observed that CAP generated a large amount of reactive oxidative species (ROS) that is the main cause of cell death. After CAP treatment a cell cycle arrest in S and G2/M phase was observed. DNA damage is observed 1 hour after treatment, suggesting this is a consequence of the treatment. They concluded that formation of DNA damages in treated cells leads to cell cycle arrest and finally to apoptosis [150]. They also conducted an in vivo experiments on U87MG (human glioblastoma cells) bearing mice and observed a significant inhibition of tumor growth (40%) at the end of the treatment compared with control group [138]. They hypothesized that DNA strand break formation mediates accumulation of tumor cells in S phase causing apoptosis in the whole tumor. It suggests that plasma components either penetrate in the tissue or induce ROS release inside of the tissue. It is encouraging, but the exact mechanism remains unclear.

Ahn et al. observed that treatment with nitrogen gas (N_2_) and air plasma jet induced apoptosis via ROS generation and dysfunction of mitochondria in human cervical carcinoma (HeLa) cells [151]. They used a plasma jet with either air or N_2_ on human cervical carcinoma HeLa cells. The cells were treated for 2 to 8 minutes. They observed N_2_ and air plasma jets induce apoptosis in a dose-dependent manner. The level of ROS increased by approximately 2-fold and 2.6-fold in HeLa cells treated with N_2_ and air plasma jets, respectively, compared with untreated cells. Interestingly, they observed depolarization of the mitochondrial membrane potential which is an early event of apoptosis. The depolarization results in mitochondrial membrane permeability, and as a result releases proapoptotic factors. They noticed a decrease of the apoptotic effect of the CAP by using scavengers of ROS. It suggests that the apoptotic effects of the plasma jet may be mediated by ROS. By using caspase-3 and caspase-9 inhibitors, they also noticed a diminution of cell death showing the potential involvement of mitochondria in apoptosis.

Panngom et al. also concluded that mitochondria may be involved in the apoptosis process following lung cancer cells exposure with non-thermal DBD plasma [147]. They observed that Mitochondrial Membrane Potential, mitochondrial enzyme activity and respiration rate were significantly decreased in cancer cells with CAP treatment compared with the normal lung cells treated with plasma. They also observed an alternation of the morphology of mitochondria.

Yan et al. proposed a mechanism of action of CAP on cancer cells in 2012 [152]. They observed CAP can control the intracellular concentrations of ROS, NO, and lipid peroxide. They showed that the concentrations of ROS, NO, and lipid peroxide are directly related to the mechanism of liver hepatocellular carcinoma (HepG2) cell death, which involves several steps. First, the plasma generates NO species, which increases the NO concentration in the extracellular medium. Then, due to a diffusion process, the intracellular NO concentration increases which leads to the increase of the intracellular ROS concentration. Finally the oxidative stress creates lipid peroxidation that injures the cell. The combined action of NO, ROS, and lipid peroxide species results in HepG2 cell death. The increased concentrations of NO, ROS, and lipid peroxide during the plasma exposure correlated with the decreasing numbers of viable cells. (See list of mechanisms of CAP on cancer cells).

#### ***List of mechanisms of CAP on cancer cells***

o Activation of p53 protein [148]

o Activation of p21 CDK inhibitor [149]

o Cell cycle arrest at the G2/M and S phase [142,149,150]

o ROS leads to DNA damages leading to cell cycle arrest [150]

o Apoptosis induced via ROS generation and dysfunction of mitochondria [151]

o Mitochondrial Membrane Potential, mitochondrial enzyme activity and respiration rate are significantly decreased in cancer cells after CAP treatment [147]

o CAP can control the intracellular concentrations of ROS, NO, and lipid peroxide [152]

The exact mechanism of action of CAP on cells still remains unclear. What cell signals CAP induces has not been clarified so far. A better understanding regarding the signaling events induced by CAP treatment on cells is required to find the optimal dose and type of plasma to be used successfully in the clinic.

## Conclusion

CAP has a bright future in dentistry and oncology due to its anti-microbial properties and its cell death properties on cells. Concerning dentistry, studies of CAP showed promising results in tooth bleaching, deactivation of biofilms in teeth, instrument sterilization, and in composite restoration. Nevertheless, progress needs to be made concerning the ideal width and depth of the plume of plasma to enable the treatment to reach lower in teeth. Promising findings obtained from in vivo and in vitro studies of CAP in oncology show that CAP will find its niche in the treatment of cancer patients in the future. However, more studies need to be performed regarding the mechanism of action.

## Abbreviations

AC: Alternative Current; APPJ: Atmospheric Pressure Plasma Jet; BLI: Bioluminescence Imaging; CAP: Cold Atmospheric Plasma; CFU: Colony Forming Unit; DC: Direct Current; DBD: Dielectric Barrier Discharge; FE-DBD: Floating Electrode Dielectric Barrier Discharge; GNP: Gold Nanoparticles; NO: Nitrogen Oxide; RF: Radio Frequency; ROS: Reactive Oxidative Species; SEM: Scanning Electron Microscopy; SMD: Surface Micro Discharge; TEM: Transmission Electron Microscopy; TMZ: Temozolomide; TTP: Tissue Tolerable Plasma.

## Competing interests

The authors declare that they have no competing interests.

## Authors’ contributions

CH identified the subject, did literature search, and wrote the initial draft. CB helped with organization and revised the manuscript. JZ-Role included review design and manuscript proof reading. All authors read and approved the final manuscript.
